# Invasive cardiac lipoma at the left ventricular intermuscular region: A case report

**DOI:** 10.3892/etm.2024.12373

**Published:** 2024-01-08

**Authors:** Juan Xia, Jian-Ping Liu, Wei Hong, Jing Ge, Yong-Heng Zhang, Lin Cao, Xian-Zheng Zhang, Xiao-Hong Chen, Qin Zhou

**Affiliations:** 1 Department of Hospital-Acquired Infection Control, Suining Central Hospital, Suining, Sichuan 629000, P.R. China; 2 Department of Cardiovascular Surgery, Suining Central Hospital, Suining, Sichuan 629000, P.R. China; 3 Intensive Care Unit, Suining Central Hospital, Suining, Sichuan 629000, P.R. China; 4 Department of Anesthesiology, Suining Central Hospital, Suining, Sichuan 629000, P.R. China; 5 Department of Operating Room, Suining Central Hospital, Suining, Sichuan 629000, P.R. China

**Keywords:** cardiac lipoma, left ventricular intermuscular, case report

## Abstract

The present study described the case of a 22-year-old woman who had symptoms of left chest pain for >6 months, with further aggravation over 2 days. Computed tomography (CT) images of the mediastinal and pulmonary windows showed low-density shadows in the left ventricle. Echocardiography indicated a slightly stronger echo cluster in the left ventricle, with a range of ~29x30x35 mm, which was closely related to the lower wall and part of the posterior wall of the left ventricle. Contrast-enhanced ultrasound showed that the left ventricular mass was enhanced in a circular and dot-line shape, with a solid mass occupying the left ventricle and a rich blood supply. CT angiography revealed a nodule of size 27x27x24 mm in the left ventricle. During the operation, it was observed that the cardiac lipoma invaded the chordae tendinae and papillary muscle, and a valve replacement was performed. Postoperative examination revealed a piece of gray and anaplastic tissue, measuring 30x22x17 mm. The pathology of the specimen showed that the morphology of the left ventricular mass met the criteria of an intramuscular lipoma. The present study reported a cardiac lipoma involving the left anterior chordae tendinae and papillary muscle, with the patient showing only nonspecific symptoms. Early surgery should be applied to improve the prognosis of cardiac lipoma.

## Introduction

Primary cardiac tumors are uncommon, with a prevalence ranging from 0.17-0.19% worldwide (1). Cardiac lipoma is a rare benign tumor that accounts for 8.4% of worldwide primary cardiac tumors (2). The symptoms of cardiac lipomas are based on their locations in the heart, although most cardiac lipomas are asymptomatic and detected incidentally (3). Cardiac lipomas originate from the subendocardium, subepicardium or myocardium, with proportions of 50, 25 and 25%, respectively (4). Moreover, they always occur in the right atrium and left ventricle (5). Studies have found that the clinical presentation of cardiac lipoma varies, ranging from mild chest discomfort to sudden death (6,7). Thus, the diagnosis and early management of cardiac lipomas are particularly important for improving the prognosis of patients.

Currently, non-invasive imaging approaches are widely used to diagnose cardiac lipomas, including computed tomography (CT), echocardiography, magnetic resonance imaging (MRI) and CT angiography (CTA) (8). Non-invasive imaging methods can also modify the treatment of cardiac lipomas. However, the diagnosis and management of cardiac lipomas are still in the exploratory stage in clinical practice, lacking a unified standard. The current study describes a cardiac lipoma that was present in the left ventricular intermuscular region using non-invasive imaging methods. In the present case, the cardiac lipoma was removed under general anesthesia intubation and cryogenic cardiopulmonary bypass, and a mechanical mitral valve replacement was performed.

## Case report

A 22-year-old woman without history of pregnancy was admitted to Suining Central Hospital (Suining, China) because of left chest pain for >6 months, with further aggravation over 2 days. The patient presented with left chest pain without obvious inducement, and the symptoms were alleviated by rest. The patient did not have a history of headache, syncope, cyanosis, finger clubbing or squatting phenomenon, and no paroxysmal dyspnea or upright breathing occurred at night. The patient visited Suining Central Hospital (Suining, China) for treatment because the left chest pain worsened and continued without relief.

Physical examination after admission was as follows: Body temperature, 36.3˚C; heart rate, 96 bpm; respiration, 20 breaths/min; blood pressure, 118/88 mmHg; height, 157 cm; body weight, 80 kg; and oxygen saturation, 99%. The thorax was normal with no tenderness in the sternum. Bilateral respiratory movement was normal, and no widening of the intercostal space was identified. In addition, bilateral tactile fremitus is normal, no pleural friction sensation, clear percussion sound on both lungs, clear breath sounds (bilateral), no dry or wet rales, and no pleural friction sound. The shape of the breasts were normal and there was no abnormal uplift of the anterior heart area or enlargement of the heart boundary. The apical area was located in the fifth intercostal area of the left midclavicular line. The cardiac rhythm was consistent, and no pathological murmurs were heard in the auscultation area of each valve.

**Figure 1:**
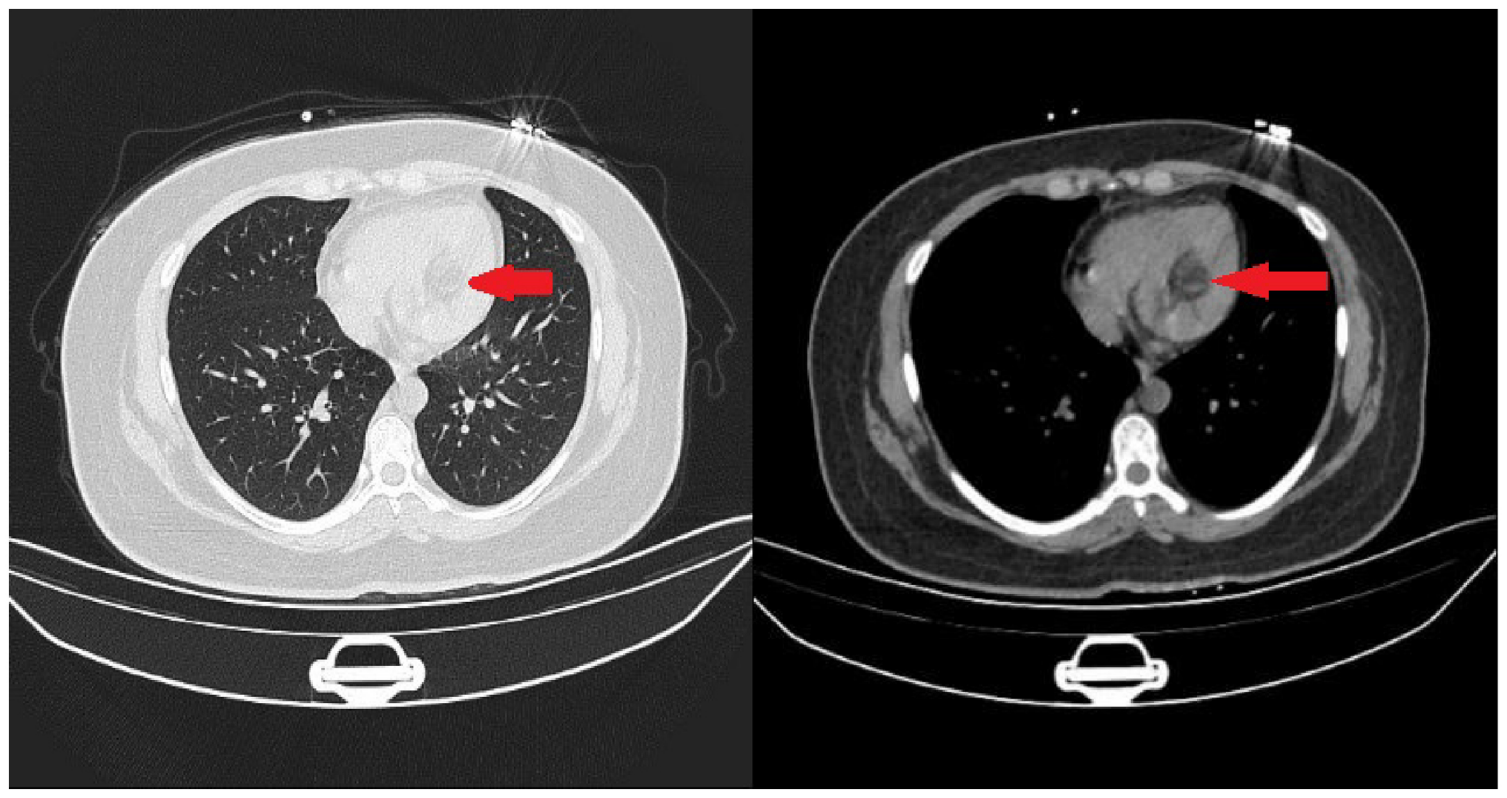
Chest computed tomography scan in the left ventricle. The arrow indicates a tumor in the left ventricle.

**Figure 2:**
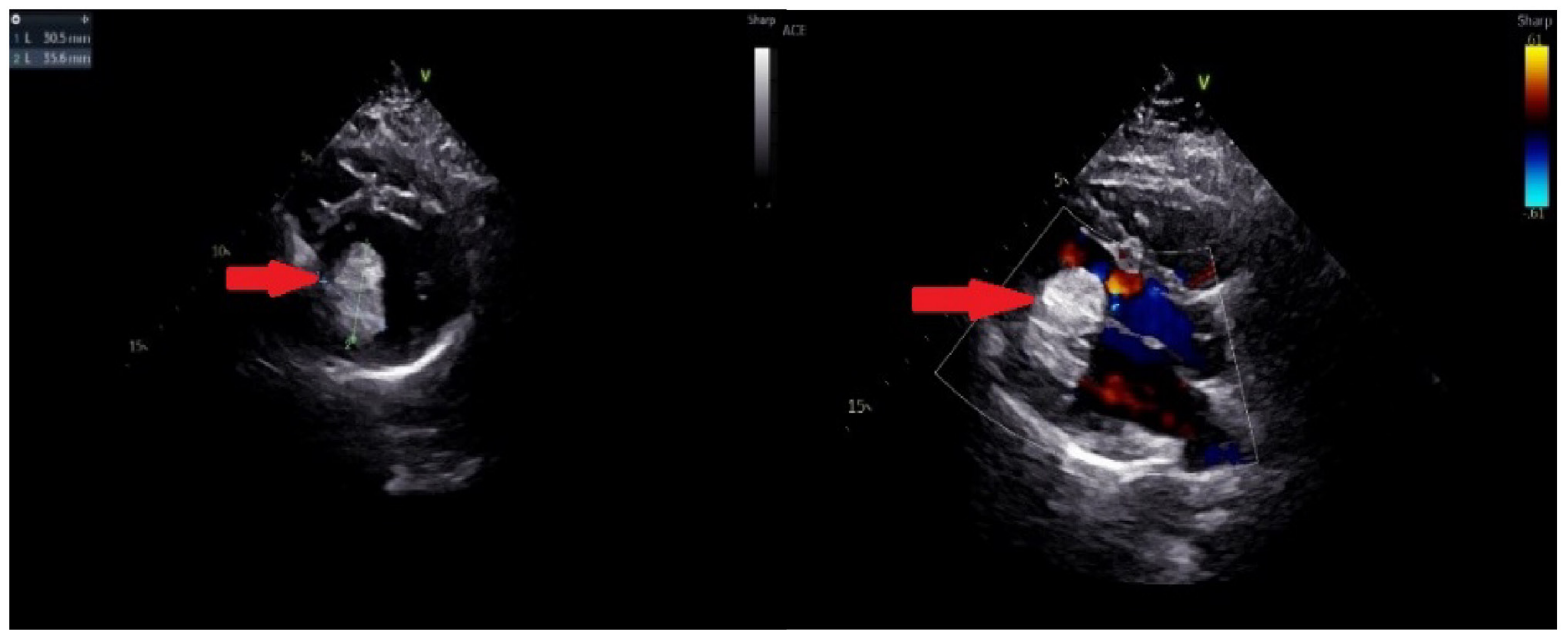
Echocardiography in the left ventricle. The arrow indicates an intracardiac tumor in the left ventricle.

**Figure 3:**
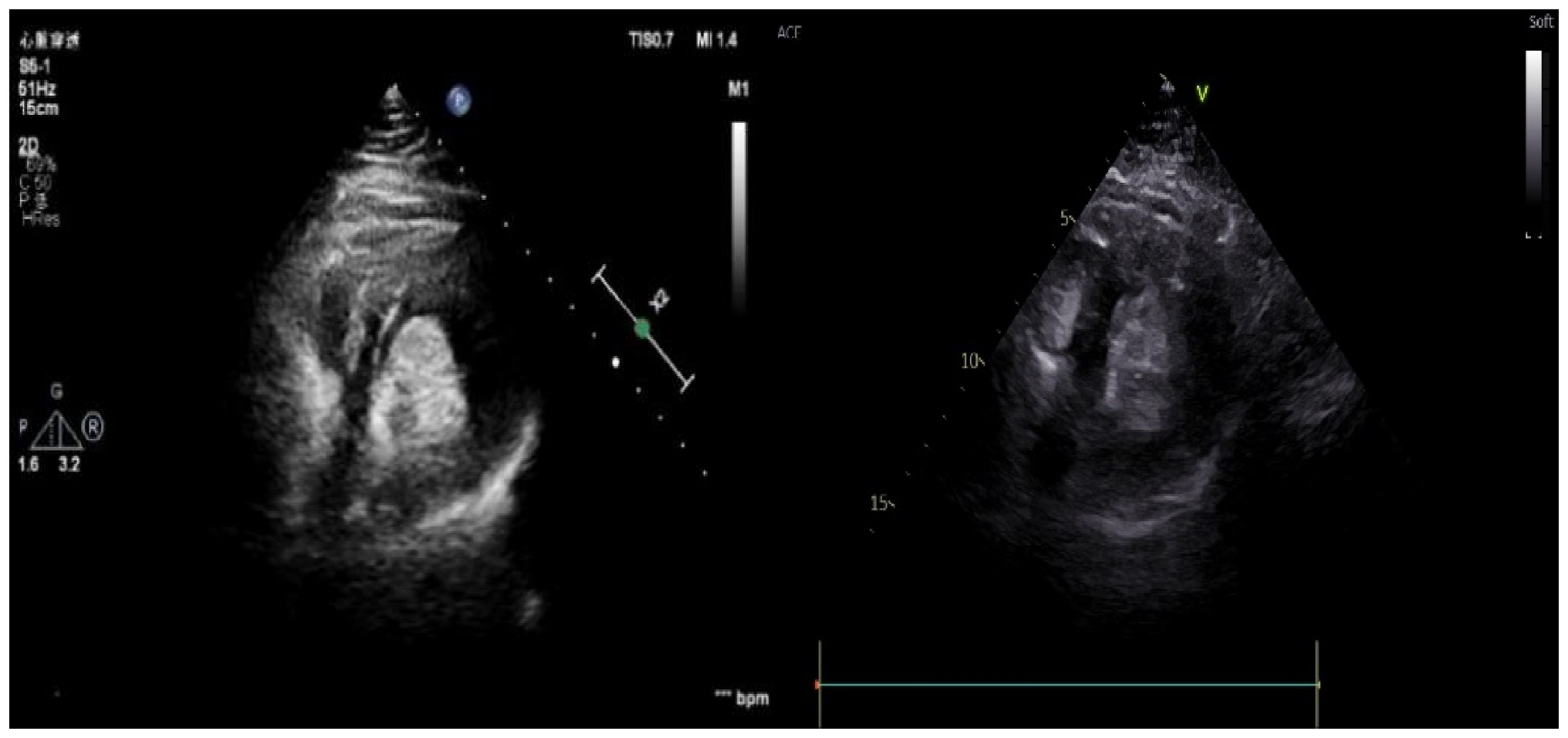
Contrast-enhanced ultrasound results.

**Figure 4:**
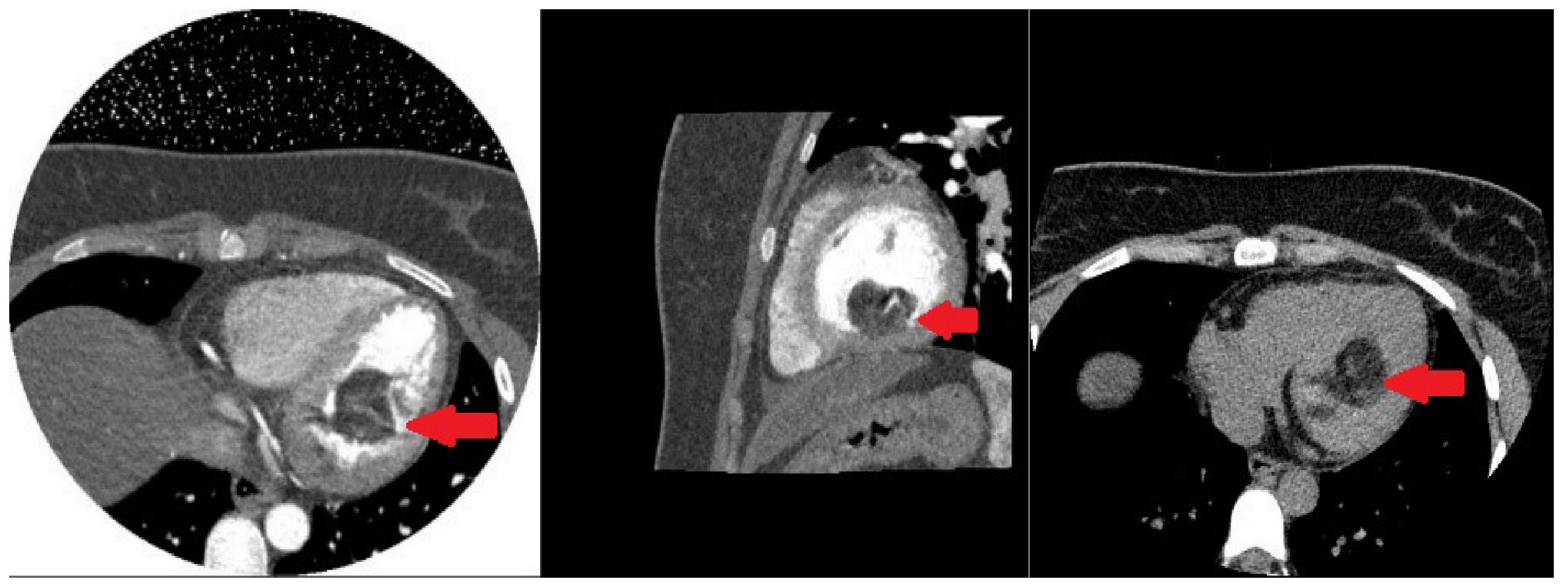
Computed tomography angiography results. The arrow indicates a tumor in the left ventricle, with a size that is partially adherent to the ventricular wall.

Electrocardiography indicated sinus tachycardia, and a chest CT scan showed a low-density shadow in the left ventricle (Fig. 1). Echocardiography also revealed a slightly stronger echo cluster in the left ventricle, with a range of ~29x30x35 mm, which was closely related to the lower wall and part of the posterior wall of the left ventricle (Fig. 2). Moreover, the preoperative ejection fraction was 60%, while the pulmonary valve velocity was 106 cm/sec. Contrast-enhanced ultrasound (CEUS) showed that the left ventricular mass was enhanced in a circular and dot-line shape, with left ventricular solid occupation and rich blood supply (Fig. 3). Moreover, CTA showed a nodule of left ventricular lipid density that was 27x27x24 mm in size (Fig. 4). Laboratory examination showed that the aspartate aminotransferase level was 45.7 U/l, while myocardial enzyme profile (creatine kinase-MB, 104.67 ng/ml; troponin I, 42433.7 pg/ml; myoglobin, 404.66 ng/ml), ESR (11 mm/h), humoral and cellular immunity (rheumatoid factors, <20.0 IU/ml; immunoglobulin G, 9.43 g/l; immunoglobulin A, 0.94 g/l; immunoglobulin M, 1.77 g/l; complement C3, 1.3 g/l; complement C4, 0.27 g/l; C-reactive protein, 5.84 mg/l) were considered normal.

**Figure 5:**
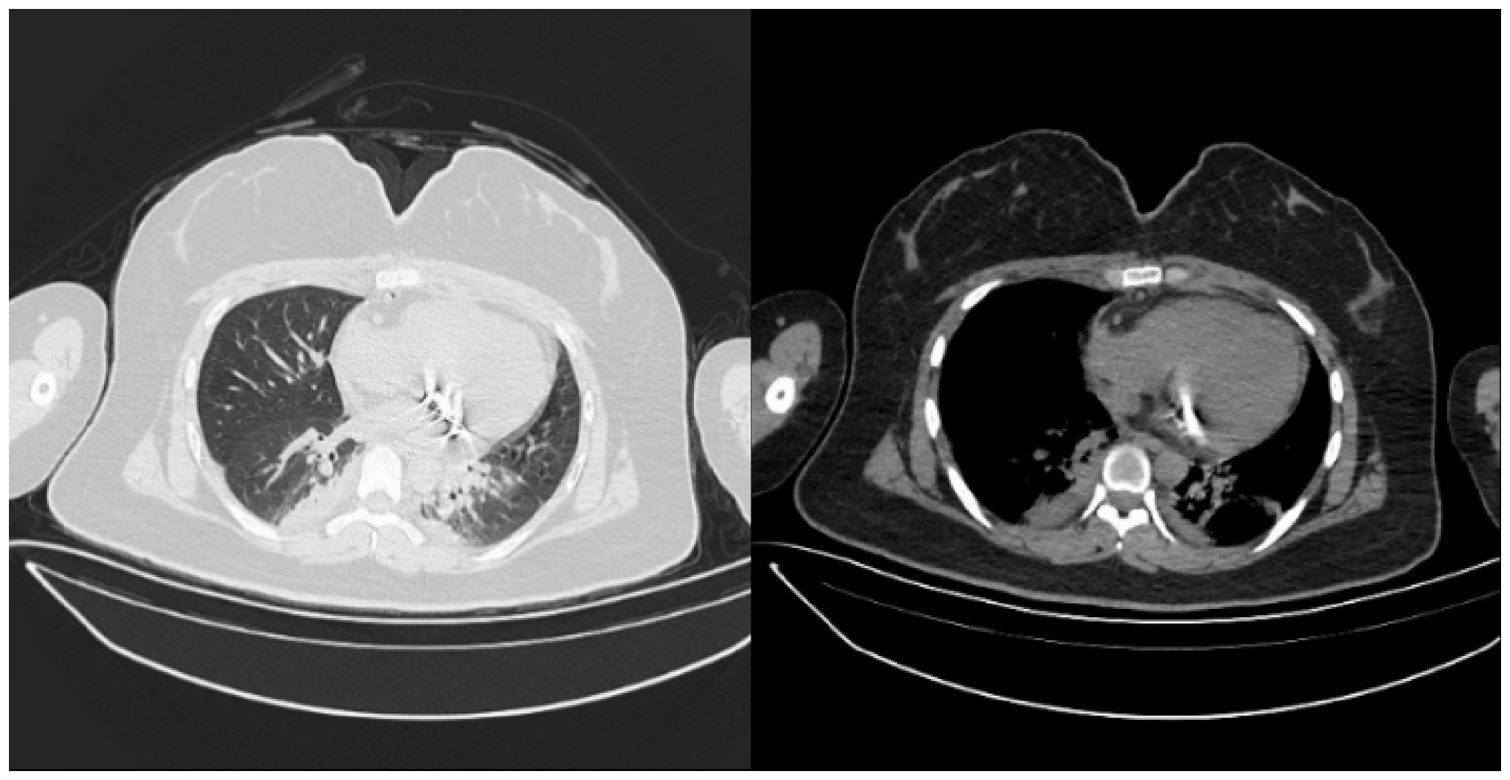
Postoperative chest computed tomography results. Complete resection of intracardiac tumor in the left ventricle, with involvement of the mitral valve. The mitral valve was removed during the surgery and replaced with a mechanical valve.

**Figure 6:**
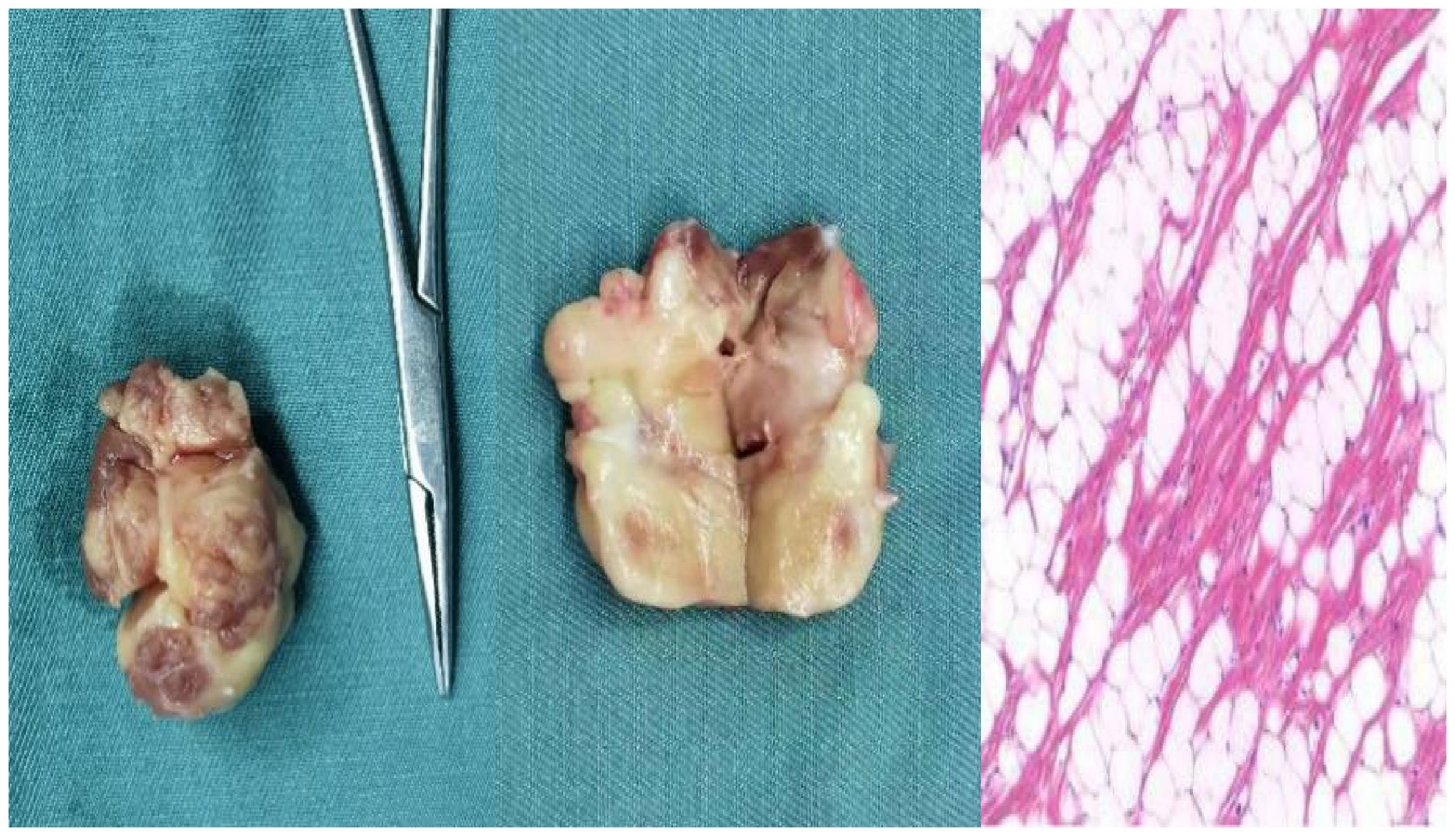
Histopathological examination of the excised mass revealed intramuscular lipoma (right panel, magnification, x50).

The heart mass was removed under general anesthesia, intubation and cryogenic cardiopulmonary bypass after preoperative preparation. An irregular grey and white mass was found in the left ventricle during the operation with a size of 30x22x17 mm, occupying ~1/3 of the space of the left ventricle. The anterior chordinae tendinae surrounding the mitral valve and the papillary muscle showed infiltrating growth. The tumor pedicle was wide and attached to the posterior inferior wall of the left ventricle, and both anterior and posterior mitral valves were normal. Considering that the nature of the mass was unknown and that the left anterior chordae tendinae and papillary muscle infiltrated and grew, complete resection of the mass could not preserve the chordae tendinae and papillary muscle. Thus, mechanical mitral valve replacement was performed simultaneously. After transition to the intensive care unit, the patient was transferred to the general ward and received routine anticoagulant treatment. All risks associated with mechanical valves, anticoagulation and pregnancy were explained to the patient prior to the procedure. Postoperative chest CT indicated that the mechanical valve was in place, and no residual tumor was identified (Fig. 5). Histopathological examination of the excised mass revealed intramuscular lipoma (Fig. 6). After 6 months, symptoms such as chest tightness had disappeared, blood coagulation function was normal and there was no evidence of tumor recurrence in the ventricle.

## Discussion

The present study reported the case of a patient with invasive cardiac lipoma in the left ventricular intermuscular region with their main symptoms including persistent left chest pain and sudden aggravation. Cardiac lipoma was diagnosed using non-invasive imaging techniques, including CT, echocardiography, CEUS and CTA. The cardiac lipoma involved the left anterior chordae tendinae and papillary muscle, and while the mass in the chordae tendinae and papillary muscle was resected, a mechanical mitral valve replacement was performed. The symptoms disappeared after the procedure, and no residual tumor was observed in the heart. To the best of our knowledge, the present study is the first to report cardiac lipoma involving the left anterior chordae tendinae and papillary muscle.

Cardiac lipomas at various locations have been reported in numerous studies; their characteristics are presented in Table I (2,4,9-39). These studies include 16 male and 17 female patients, and they have no obvious difference in sex. Moreover, the ages of the included patients ranged from 0.1-77.0 years, while the majority of patients (25/33) were aged ≥30.0 years. Most cardiac lipomas are asymptomatic and have a good prognosis; however, some giant cardiac lipomas can cause left ventricular inflow or outflow disturbances, left ventricular dysfunction or conduction system invasion (30,40). These conditions can induce dyspnea, presyncope, syncope or palpitations (30,40); however, the present study reported on a patient that presented with no etiologic chest pain, which could be considered as cardiac lipoma in clinical practice. Cardiac lipomas can be located in any part of the heart and typically originate from the epicardial fat or pericardial fat. They are surrounded by fibrous tissue and contain a small amount of myocardial tissue, as well as components of connective tissue (31). Lipomas located in the myocardium are usually small with a complete capsule, and occasionally grow on the mitral or tricuspid valve (31). The most common location is the atrial septum, followed by the endocardium of the right atrium and left ventricle (41), while in a few cases the lipomas are located in the myocardium, subepicardium and pericardium (29). In the present study, an invasive cardiac lipoma was observed in the left ventricular intermuscular region.

Echocardiography is the initial choice for the diagnosis of cardiac lipomas, but it cannot detect smaller tumors (30). Moreover, echocardiography cannot provide clear information regarding lipomas and other primary cardiac tumors (42). Thus, CT and MRI should be performed to provide useful information on tissue characteristics and the degree of myocardial infiltration (30,41). Especially for MRI, which is considered to be the most accurate diagnostic tool owing to their distinctive fatty characteristics, they can be easily identified as masses (41). A previous study has demonstrated that lipomas show up clearly in T1- and T2-weighted images, accompanied with complete suppression on fat-saturated sequences (43). In the present study, the patient had repeated chest tightness, chest pain and discomfort, accompanied by palpitations, which might be related to outflow tract obstruction caused by a left ventricular tumor or paroxysmal ventricular tachycardia caused by conduction tissue compression; this was not discovered until B-mode ultrasound was performed. Furthermore, CT indicated the presence of dense lipid nodules, which supported evidence of a lipoma, further suggesting that CT imaging can be used to differentiate the properties of a tumor.

Considering that sudden death risk has already been reported in patients with cardiac lipomas, surgical resection should be performed irrespective of the symptoms of cardiac lipomas (44). The surgical method of cardiac lipoma resection is usually performed through a median sternum incision under complete cardiopulmonary bypass (44). Cardiac lipomas are always encapsulated, rarely invade the heart muscles and are easy to remove (44). Additionally, early surgical resection of small lipomas can preserve heart function (44); however, conduction dysfunction caused by lipoma invasion of the heart muscle can lead to arrhythmias, which is the most common complication after surgical removal of cardiac tumors (45). In the present case, the lipoma infiltrated the anterior chordae tendinae surrounding the mitral valve and papillary muscle, and the scope of infiltration growth was small. Heart integrity might not be affected after complete resection of the tumor (44); thus, complete resection was selected to prevent postoperative recurrence risk. Simultaneous mitral valve replacement may be a viable surgical option for patients with complete resection of the mass and no retention of the chordae tendinae or papillary muscle.

Left ventricular infiltrating lipoma is a rare disease; however, owing to the size and location of the tumor, corresponding obstruction symptoms or conduction disorders caused by invasion of the myocardium may lead to arrhythmias (7). Furthermore, the myocardial tissue in the corresponding section becomes thinner. Therefore, in asymptomatic young patients presenting with the aforementioned electrocardiographic manifestations, echocardiography should be performed to avoid misdiagnosis. Surgical treatment is the preferred treatment for cardiac lipoma, and complete resection is still a viable treatment option when the lesion scope is small for invasive lipomas. If the lesion involves the valve, chordae tendinae, papillary muscle or other tissues, heart valve replacement should be performed.

## Figures and Tables

**Table I:** Summary results for patients with cardiac lipoma.

First author/s, year	Age, years	Sex	Diagnostic tool	Tumor location	(Refs.)
Kamiya *et al*, 1990	45.0	Male	Echocardiography, CT, MRI, CTA	Interventricular septum	(9)
Morikami *et al*, 1994	38.0	Male	Echocardiography, CT, MRI	Inferior wall of the left ventricle near the apex	(10)
Fukushima *et al*, 1999	22.0	Male	Echocardiography, MRI	Left ventricle beneath the mitral valve	(11)
Schrepfer *et al*, 2003	31.0	Female	Echocardiography, CT, MRI, CTA	Right ventricular	(12)
Agacdiken *et al*, 2005	18.0	Female	Chest X-ray, echocardiography, CT, MRI	Interventricular septum	(13)
Friedberg *et al*, 2006	13.0	Male	Chest X-ray, echocardiography, MRI	Lateral mitral annulus	(14)
Ozaki *et al*, 2006	74.0	Female	Chest X-ray, echocardiography, CT, MRI	Interventricular septum	(15)
Arslan *et al*, 2007	45.0	Male	Echocardiography, CT	Interventricular septum	(16)
Ganame *et al*, 2008	62.0	Male	Echocardiography, MRI	Endocardial surface of the mid-anterior septum	(17)
Hayashi *et al*, 2008	55.0	Male	Echocardiography, CT	Left ventricular myocardium	(18)
Gan *et al*, 2008	0.1	Male	Echocardiography, CT	Right atrium	(19)
Kawarai *et al*, 2010	57.0	Male	Echocardiography, CT, MRI	Left ventricular	(20)
Song *et al*, 2010	56.0	Female	Chest X-ray, echocardiography, CT, CTA	Aneurysmal right ventricle	(21)
Domoto *et al*, 2010	70.0	Male	Echocardiography, CT, MRI	Left ventricular apex	(22)
Frank *et al*, 2012	36.0	Female	Echocardiography	Anterior wall	(23)
Schiettecatte *et al*, 2012	68.0	Female	Echocardiography, MRI	Intracardiac	(24)
Singh *et al*, 2013	26.0	Female	Echocardiography, MRI	Mimicking atrial myxoma	(25)
Tanaka *et al*, 2015	77.0	Female	Echocardiography, CT, MRI	Left ventricular apex	(26)
Li *et al*, 2015	65.0	Male	Echocardiography, CT, MRI	Interventricular septum	(2)
Shenthar *et al*, 2015	25.0	Male	Echocardiography, CT, MRI	Right ventricle	(27)
Zhang *et al*, 2016	49.0	Female	Echocardiography, MRI	Right ventricular	(28)
Fang *et al*, 2016	48.0	Female	Echocardiography, MRI	Right ventricle	(29)
D'Souza *et al*, 2017	33.0	Male	Echocardiography, MRI	Right atrium	(4)
Sun *et al*, 2018	70.0	Female	Echocardiography, MRI	Left ventricular	(30)
Karangelis *et al*, 2019	72.0	Female	Echocardiography, MRI	Right ventricle	(31)
Shamsi *et al*, 2020	57.0	Male	Echocardiography, MRI	Left ventricular	(32)
Elenizi *et al*, 2020	17.0	Female	Echocardiography, CT, MRI	Right ventricular	(33)
Abdelradi *et al*, 2020	51.0	Male	Echocardiography, CT, MRI	Left ventricular	(34)
Bai *et al*, 2021	25.0	Female	Echocardiography, CT, MRI	Right atrium	(35)
Younes *et al*, 2021	49.0	Female	Echocardiography, MRI	Left ventricular	(36)
Nepal *et al*, 2022	50.0	Female	Echocardiography, CT, MRI	Interventricular septum	(37)
Watanabe *et al*, 2022	51.0	Female	Echocardiography, CT, MRI	Posterior surface of the heart	(38)
Bajdechi *et al*, 2022	30.0	Male	Echocardiography, MRI	Right atrium	(39)

CT, computed tomography; MRI, magnetic resonance imaging; CTA, CT angiography.

## Data Availability

The datasets used and/or analyzed during the current study are available from the corresponding author on reasonable request.

## References

[1] Wu S, Teng P, Zhou Y, Ni Y (2015). A rare case report of giant epicardial lipoma compressing the right atrium with septal enhancement. J Cardiothoracic Surg.

[2] Li D, Wang W, Zhu Z, Wang Y, Xu R, Liu K (2015). Cardiac lipoma in the interventricular septum: A case report. J Cardiothoracic Surg.

[3] McAllister HAJ, Fenoglio JJJ, Fine G

[4] D'Souza J, Shah R, Abbass A, Burt JR, Goud A, Dahagam C (2017). Invasive cardiac lipoma: A case report and review of literature. BMC Cardiovasc Disord.

[5] Ismail I, Al-Khafaji K, Mutyala M, Aggarwal S, Cotter W, Hakim H, Khosla S, Arora R (2015). Cardiac lipoma. J Community Hosp Intern Med Perspect.

[6] Puvaneswary M, Edwards JR, Bastian BC, Khatri SK (2000). Pericardial lipoma: Ultrasound, computed tomography and magnetic resonance imaging findings. Australas Radiol.

[7] Li J, Ho SY, Becker AE, Jones H (1998). Multiple cardiac lipomas and sudden death: A case report and literature review. Cardiovasc Pathol.

[8] Monti L, Scardino C, Nardi B, Balzarini L (2015). Lipoma of the interventricular septum. Eur Heart J.

[9] Kamiya H, Ohno M, Iwata H, Ohsugi S, Sawada K, Koike A, Ogawa K, Yano Y, Hayase S, Horiba M (1990). Cardiac lipoma in the interventricular septum: Evaluation by computed tomography and magnetic resonance imaging. Am Heart J.

[10] Morikami Y, Higashi T, Isomura T, Hirano A, Tanaka K, Hisatomi K, Ohishi K (1994). Cardiac lipoma with changes of ST segment and T wave on electrocardiogram. Jpn Circ J.

[11] Fukushima KK, Mitani T, Hashimoto K, Hosogi S, Emori T, Morita H, Fujimoto Y, Nakamura K, Yamanari H, Ohe T (1999). Ventricular tachycardia in a patient with cardiac lipoma. J Cardiovasc Electrophysiol.

[12] Schrepfer S, Deuse T, Detter C, Treede H, Koops A, Boehm DH, Willems S, Lacour-Gayet F, Reichenspurner H (2003). Successful resection of a symptomatic right ventricular lipoma. Ann Thorac Surg.

[13] Agacdiken A, Gurbuz Y, Ciftci E, Omay O, Vural A, Ural D (2005). Cardiac lipoma in a patient with proven arrhythmogenic right ventricular dysplasia: A case report. A huge intramyocardial lipoma. Int J Cardiovasc Imaging.

[14] Friedberg MK, Chang IL, Silverman NH, Ramamoorthy C, Chan FP (2006). Images in cardiovascular medicine. Near sudden death from cardiac lipoma in an adolescent. Circulation.

[15] Ozaki N, Mukohara N, Yoshida M, Shida T (2006). Cardiac lipoma in the ventricular septum-a case report. Thorac Cardiovasc Surg.

[16] Arslan S, Gundogdu F, Acikel M, Kantarci AM (2007). Asymptomatic cardiac lipoma originating from the interventricular septum diagnosed by multi-slice computed tomography. Int J Cardiovasc Imaging.

[17] Ganame J, Wright J, Bogaert J (2008). Cardiac lipoma diagnosed by cardiac magnetic resonance imaging. Eur Heart J.

[18] Hayashi H, Hidaka F, Kiriyama T, Sato H, Takagi R, Kumita S (2008). A left ventricular lipoma diagnosed on three-dimensional electrocardiogram-gated cardiac computed tomography. Heart Vessels.

[19] Gan C, An Q, Tao K, Tang H, Li W (2008). An asymptomatic lipoma of the right atrium in a neonate. J Pediatr Surg.

[20] Kawarai S, Yaginuma GY, Abe K, Hamasaki A, Ishikawa K, Tanaka D (2010). Left ventricular lipoma with pseudoaneurysm-like appearance. Gen Thorac Cardiovasc Surg.

[21] Song Y, Hickey W, Nabi F, Chang SM (2010). Extensive cardiac lipoma with aneurysmal right ventricle. Interact Cardiovasc Thorac Surg.

[22] Domoto S, Nakano K, Kodera K, Sasaki A, Asano R, Ikeda M, Kataoka G (2010). Cardiac lipoma originating from the left ventricular apex diagnosed using the magnetic resonance imaging fat suppression technique: Report of a case. Surg Today.

[23] Frank S, Pochmalicki G, Achor A, Debauchez M, Ha DE (2012). Successful resection of an intra-cardiac lipoma during the first trimester of pregnancy, coming to term normally. Eur J Obstet Gynecol Reprod Biol.

[24] Schiettecatte A, Verdries D, de Mey J, De Maeseneer M, Dujardin M (2012). Magnetic resonance imaging findings in cardiac lipoma. JBR-BTR.

[25] Singh B, Bhairappa S, Shankar SK, Prasad NM, Manjunath CN (2013). Cardiac lipoma at unusual location-mimicking atrial myxoma. Echocardiography.

[26] Tanaka Y, Yoshimuta T, Yamagishi M, Sakata K (2015). Video-assisted transmitral resection of primary cardiac lipoma originated from the left ventricular apex. Eur Heart J Cardiovasc Imaging.

[27] Shenthar J, Sharma R, Rai MK, Simha P (2015). Infiltrating cardiac lipoma presenting as ventricular tachycardia in a young adult. Indian Heart J.

[28] Zhang HW, Zhong MH, Meng W, Zhang EY, Gu J, Hu J (2016). Intramuscular lipoma as an unusual cause of right ventricular outflow tract obstruction. Echocardiography.

[29] Fang L, He L, Chen Y, Xie M, Wang J (2016). Infiltrating lipoma of the right ventricle involving the interventricular septum and tricuspid valve: Report of a rare case and literature review. Medicine (Baltimore).

[30] Sun X, Liu G, Kim H, Sun W (2018). Left ventricular lipoma resected using thoracoscope-assisted limited sternotomy: A case report and literature review. Medicine (Baltimore).

[31] Karangelis D, Palios J, Tzertzemelis D, Economidou S, Panagiotou M (2019). Surgical resection of a cardiac lipoma of the right ventricle. Ann Card Anaesth.

[32] Shamsi F, Bajwa G, Ghalib H (2020). ‘Left ventricular lipoma….. a rare case’, case report. J Cardiothorac Surg.

[33] Elenizi K, Matta A, Alharthi R, Campelo-Parada F, Lhermusier T, Bouisset F, Elbaz M, Carrié D, Roncalli J (2020). Incidental discovery of right ventricular lipoma in a young female associated with ventricular hyperexcitability: An imaging multimodality approach. World J Cardiol.

[34] Abdelradi A, Yekta A (2020). A case of asymptomatic cardiac lipoma and literature review. CJC Open.

[35] Bai R, Zhang Y, Wang H, Yang J, Sun D (2021). Invasive cardiac lipoma diagnosis based on echocardiography: Case report and literature review. J Clin Ultrasound.

[36] Younes A, Ahmad S, Yousaf A, Marcu CB (2021). A rare presentation of cardiac lipoma as an acute coronary syndrome: A case report and review of literature. Cureus.

[37] Nepal S, Deshmane SB, Donovan K, May A, Chaudhuri D (2022). Invasive lipoma of the interventricular septum, a rare benign cardiac mass with atypical presentation and management. J Investig Med High Impact Case Rep.

[38] Watanabe S, Ichihara Y, Morita K, Saito S, Niinami H (2022). Successful removal of a posterior cardiac lipoma by transection of the ascending aorta and main pulmonary artery. Case Rep Cardiol.

[39] Bajdechi M, Onciul S, Costache V, Brici S, Gurghean A (2022). Right atrial lipoma: A case report and literature review. Exp Ther Med.

[40] Lin HD, Hsu PF, Wu MH, Leu HB, Hsu TL (2006). Images in cardiology: Subaortic stenosis caused by left ventricular outflow tract lipoma. Clin Cardiol.

[41] Rocha RV, Butany J, Cusimano RJ (2018). Adipose tumors of the heart. J Card Surg.

[42] Barbuto L, Ponsiglione A, Del Vecchio W, Altiero M, Rossi G, De Rosa D, Pisani A, Imbriaco M (2015). Humongous right atrial lipoma: A correlative CT and MR case report. Quant Imaging Med Surg.

[43] Hoey ET, Mankad K, Puppala S, Gopalan D, Sivananthan MU (2009). MRI and CT appearances of cardiac tumours in adults. Clin Radiol.

[44] D'Errico S, Mazzanti A, Frati P, Fineschi V (2019). Conduction disorder and primary cardiac tumor: A fatal case of multiple lipomas of the right atrium. J Geriatr Cardiol.

[45] Li S, Gao C (2017). Surgical experience of primary cardiac tumor: Single-institution 23-year report. Med Sci Monit.

